# Improving the indicator for premature deaths from noncommunicable diseases

**DOI:** 10.2471/BLT.20.254110

**Published:** 2020-05-01

**Authors:** Shah Ebrahim, Pedro Ordunez, Peter Lloyd-Sherlock, Martin McKee, Ramon Martinez, Patricia Soliz

**Affiliations:** a Department of Non-communicable Disease Epidemiology, London School of Hygiene and Tropical Medicine, Keppel Street, London WC1E 7HT, England.; b Pan American Health Organization, Washington, DC, United States of America.; c School of International Development, University of East Anglia, Norwich, England.

Indicators that monitor health policies and programmes reflect the strategic foundations of pursued goals and guide interventions and are therefore critical to public health. Hence, using premature mortality (that is, deaths among people 30–69 years of age) from selected noncommunicable diseases as an indicator for monitoring the health-related sustainable development goal (SDG) target of reducing noncommunicable disease premature mortality by a third by 2030, has been disputed as being ageist and for focusing on only four groups of diseases.^1^^–^^3^ Rebuttal to this criticism is based on assertions that premature mortality correlates closely with life expectancy, is easy to understand, is consistent with the World Health Assembly 2012 resolution WHA65.3 on noncommunicable diseases, and is the global priority for these diseases.^4^

Ageism is a value set rather than an epidemiological concept and engenders attitudes that reinforce stereotypes of older people as frail and of low social and economic value. We argue that this health target indicator, based on the existing definition of premature mortality, could be perceived as ageist. Our argument is based on ethical principles, recognition of the achievements of age-based health care in wealthier countries and evidence of cost–effective benefits of prevention and treatment for noncommunicable diseases in older people.^1^^,^^3^ Furthermore, we are concerned about the semantic and intuitive effects of age-bounded definitions of premature mortality. Describing avoidable deaths from noncommunicable diseases of people older than 69 years of age as non-premature leads to reinforcement and justification of the ageist bias that often permeates the health sector. The article from Byass^5^ illustrates this ageist bias by showing the correlation between mortality rates from noncommunicable diseases at 30 to 69 years of age with those 70 to 89 years of age, and concluding that the premature mortality indicator, therefore, should not be construed as ageist.^5^ Mortality rates for different age groups are correlated because time and geographic trends in noncommunicable disease mortality are largely due to period effects, with cohort effects playing a less important role. Byass’ finding is not inevitable. For example, correlations may change as the smoking epidemic in low- and middle-income countries impacts progressively on older age groups. The key difference between mortality rates in people younger than 70 years of age and those older is their increased order of magnitude: 3.9 per 100 000 versus 43 per 100 000. Although the rates correlate, using an indicator that includes the age groups that have the major burden of disease would be better. 

The target indicator also applies a restricted definition of noncommunicable diseases, cardiovascular disease, cancers, diabetes and chronic respiratory disease. Byass notes that this limited definition is often justified by the difficulty of ascertaining cause of death in older people.^5^ However, the undetermined causes of death by age group reported were actually small, 14.6% (4364/29 911) among those older than 65 years of age compared with 11.5% (782/68 518) younger than 65 years of age, indicating that ascertainment of cause of death is not a barrier to expanding the noncommunicable diseases included in an SDG mortality indicator.

In our analysis of cardiovascular disease mortality from 36 countries of the WHO region of the Americas, people 70 years of age or older accounted for the majority of cardiovascular disease deaths in all these countries, ranging from 52.0% in the Bahamas to 80.0% in Canada.^6^ Furthermore, between 2000 and 2015, the annual average percentage changes in cardiovascular disease mortality in Brazil, Canada, Colombia, Mexico and the United States of America generally decreased, with the exception of Mexico, and the largest reductions were observed in age groups older than 70 years of age. The proportion of the population older than 70 years of age is estimated to double from 5.6% in 2000 to 10.5% by 2030,^7^ which gives further motivation to use an indicator capable of capturing trends among older people.

Byass further justified the SDG indicator by stating that reducing the probability of noncommunicable disease deaths at older ages is less feasible than among those 30 to 69 years of age.^5^ However, extensive evidence exists that many preventive and health-care interventions are of greater benefit at older ages in terms of absolute rate reductions in mortality and in cost–effectiveness.^3^

## Noncommunicable disease selection

The noncommunicable disease countdown 2030 group is concerned that the SDG indicator only partially captures global inequalities and should be more comprehensive and inclusive.^2^ The group has proposed using a different outcome alongside monitoring progress towards the related SDG target: all deaths in people younger than 80 years of age from noncommunicable diseases. The group’s analyses showed that annual time trends for the SDG indicator and the comprehensive noncommunicable disease outcome were highly correlated. The SDG indicator gave a more optimistic picture of progress than the comprehensive outcome in many populations. The target will be achieved in 35/188 countries (19%) for women, and 30/188 (16%) for men, if these countries maintain or surpass their 2010–2016 rate of decline in noncommunicable disease mortality.^8^ If the comprehensive outcome were to be adopted as the indicator, only 17/188 countries (9%) for women and 5/188 countries (3%) for men would be on track to reduce the probability of death by a third by 2030.^2^ This difference is probably explained by the comprehensive outcome having a greater proportion of noncommunicable disease deaths that are not avertable by personal health care, public health programmes or policies.

## Current indicator consequences

Byass further asserts that the use of this indicator neither involves nor implies any lack of care or concern for older people.^5^ However, excluding older people from the indicator creates an incentive for countries with no civil registration and vital statistics systems and limited resources to prioritize collecting cause of death data only for those 30–69 years of age, which is clearly an incomplete and limited approach. The link between an SDG indicator and the relevant health-care delivery seems obvious; if the former is selective, the latter will likely be too. Health targets are intended to drive health policy and services, which are embedded in the drive towards universal health coverage (UHC). However, the tracer interventions specified for this goal do not include any noncommunicable disease interventions and are limited to targets from the millennium development goals (immunization, maternal-child health, human immunodeficiency virus, tuberculosis and insecticide-treated bednets).^8^

Including tracer interventions for noncommunicable diseases in the UHC goal would improve the likelihood that services would be developed, and deaths and long-term, serious disability would be averted. Our inability to manage the societal costs of noncommunicable diseases in older people is now obvious in high-income countries. In low- and middle-income countries, incorporating health and social care for ageing populations into health policy is critical. A recent evaluation of SDG impacts concluded that all led to unintended consequences for other important objectives and development.^9^ Many low- and middle-income countries are dependent on development assistance for funding health care. Such funds are largely spent on younger people and only 2.3% of these funds are directed towards noncommunicable diseases.^10^ Chronic diseases that typically affect older people receive only 0.3% of 36.4 billion United States dollars in 2015 of development funds for health. Since 1990, the funds for development assistance have quadrupled but the proportion of funding on noncommunicable diseases has declined despite the rising burden of these diseases.^10^ Health care for older people with noncommunicable disease could be more comprehensive if international and national targets and indicators focused on matching development funding on the burden of disease. 

## An alternative approach

Both the current indicator and the 2030 countdown group’s approach are capable of generating useful data for assessing progress but we believe these indicators can be improved. Our current work has involved using existing lists of noncommunicable disease causes of death that are either preventable through public health policies and interventions or amenable to health care to create a list of avertable noncommunicable disease causes of death, which was then mapped to the Global Burden of Disease (GBD) cause list. Age-standardized years of life lost (YLL) per 100 000 population due to avertable and non-avertable mortality from noncommunicable diseases causes by sex, location and year were estimated from the GBD study. Trends in age-standardized YLL due to avertable and non-avertable such diseases from 1990 to 2017 were examined to assess the progress of avertable mortality from these diseases in achieving the health target on noncommunicable diseases.^11^ We have termed the indicator premature avertable mortality from noncommunicable diseases – premature in relation to the gap between age at death and expected life expectancy at that age. Our findings are that the indicator fell in every WHO region and in most countries and territories between 1990 and 2017. Despite these reductions, only the WHO Western Pacific and European Regions and 25 countries (most of which are high-income countries) are on track to achieve this SDG target. Since 2013, there has been a global slowdown in the reduction of premature avertable mortality from noncommunicable diseases and a stagnation in the WHO Region of the Americas.

Measuring such mortality gives comparisons of countries’ progress in preventing and delaying deaths from conditions amenable to public health interventions and health care. This goal and measure should spur countries that are currently off track to re-evaluate and strengthen their action plans, including those for universal health coverage.
